# The Importance of Semantic Network Brain Regions in Integrating Prior Knowledge with an Ongoing Dialogue

**DOI:** 10.1523/ENEURO.0116-22.2022

**Published:** 2022-09-19

**Authors:** Petar P. Raykov, James L. Keidel, Jane Oakhill, Chris M. Bird

**Affiliations:** School of Psychology, University of Sussex, Falmer BN1 9QH, United Kingdom

**Keywords:** fMRI, memory, prior knowledge, semantic

## Abstract

To understand a dialogue, we need to know the topics that are being discussed. This enables us to integrate our knowledge of what was said previously to interpret the current dialogue. This study involved a large-scale behavioral experiment conducted online and a separate fMRI experiment, both testing human participants. In both, we selectively manipulated knowledge about the narrative content of dialogues presented in short videos. The clips were scenes from situation comedies that were split into two parts. The speech in the part 1 clips could either be presented normally or spectrally rotated to render it unintelligible. The part 2 clips that concluded the scenes were always presented normally. The behavioral experiment showed that knowledge of the preceding narrative boosted memory for the part 2 clips as well as increased the intersubject semantic similarity of recalled descriptions of the dialogues. The fMRI experiment replicated the finding that prior knowledge improved memory for the conclusions of the dialogues. Furthermore, prior knowledge strengthened temporal intersubject correlations in brain regions including the left angular gyrus and inferior frontal gyrus. Together, these findings show that (1) prior knowledge constrains the interpretation of a dialogue to be more similar across individuals; and (2), consistent with this, the activation of brain regions involved in semantic control processing is also more similar between individuals who share the same prior knowledge. Processing in these regions likely supports the activation and integration of prior knowledge, which helps people to better understand and remember dialogues as they unfold.

## Significance Statement

Understanding a conversation involves knowing what was previously said, especially when picking up a conversation after a break. Here we manipulated prior knowledge about the topic of conversation while participants watched dialogues between two people. Participants watched video clips for which they either had heard the preceding dialogue normally or as incomprehensible jumbled speech. Even in the conversations with jumbled speech, the general situation was clear (where it was set, who was there, and what they were doing). Prior knowledge resulted in better memory for the dialogues and also more similar recalled descriptions of the conversations across participants. Furthermore, the coherence of brain activity in language regions was increased across participants who shared the same prior knowledge.

## Introduction

Understanding an unfolding narrative, such as a conversation from a television show, requires multiple inter-related cognitive processes, yet it is something that we typically accomplish with remarkable ease (Lee et al., 2020). In addition to processing the speech, gestures, and expressions of the speakers, we need to activate relevant prior knowledge to understand what is currently being discussed. This is essential in the situation where a dialogue is resumed after a break; to pick up the conversation, we need to remember exactly what was being discussed. In this study, we investigate how knowledge of the earlier conversation affects comprehension and memory for dialogues between people. More generally, we examine the brain regions that integrate prior knowledge from long-term memory with incoming narrative information.

A fruitful method of investigating the brain regions engaged during relatively lifelike situations is to scan participants while they listen to stories, or watch video clips, and measure the degree to which brain activity is synchronized across individuals [the “intersubject correlation” (ISC); Nummenmaa et al., 2018; Nastase et al., 2019]. This technique has been successfully used to investigate how prior knowledge impacts the processing of narrative material. Some studies have directly manipulated prior knowledge by providing information that enables the participants to better understand what is happening (van Kesteren et al., 2010; Ames et al., 2015; Chen et al., 2016; Heidlmayr et al., 2020), or to interpret the narrative in a specific way (Lahnakoski et al., 2014; Yeshurun et al., 2017). Other studies have relied on pre-existing differences between participants, which meant that they are likely to process the narratives in more similar ways, such as people who are closer in a social network (Parkinson et al., 2018), have a similar personality trait (Finn et al., 2018), or support the same sports team (Andrews et al., 2019). Finally, other studies have used ambiguous narratives and have correlated neural similarity with the specific interpretation made by participants (Nguyen et al., 2019; Saalasti et al., 2019).

In general, these previous studies converge to show that any manipulation that aligns the interpretation of a narrative across participants increases ISC of brain activity in higher-order “association” regions, especially those associated with the default mode network (DMN; Raichle et al., 2001) and the “social brain” network (Alcalá-López et al., 2018). It is assumed that the increased synchrony in those regions reflects the adoption of more similar cognitive task sets and knowledge schemas when participants process the stimuli.

In a previous study, Keidel et al. (2018) proposed a more specific role for regions commonly identified in studies of narrative processing, namely the angular gyrus (AG), middle temporal gyrus (MTG), and inferior frontal gyrus (IFG). They suggested that these regions play a central role in representing the semantic concepts necessary to understand a conversation. The study manipulated knowledge about scenes taken from television situation comedies (henceforth, “TV sitcoms”), which showed conversations between the characters. The scenes were split into two parts. In one condition, the part 2 clips were straightforward completions of the part 1 clips, which could be easily understood with reference to the beginning of the conversation. In the second condition, the two clips were taken from the same show, but a different episode, so while the characters and locations were the same, the topic of the conversation was completely different across the two clips. Clips that were straightforward completions—where participants had knowledge of the preceding conversation—were associated with higher activity in the AG, MTG, and IFG.

Recently, studies of narrative processing have benefited from advances in automated techniques for quantifying the similarity of text (words or sentences) within a multidimensional semantic space [e.g., Word2Vec and the Google Universal Sentence Encoder (USE); Cer et al., 2018]. For example, Heusser et al. (2021) converted the narrative content of a movie to trajectories through a semantic space, and then compared these to brain activity to identify regions that are sensitive to semantic changes in an ongoing narrative. Another study showed that higher-order brain regions preferentially represent meaning at the level of topics of whole sentences rather than at the level of individual words (Acunzo et al., 2022). Recently, Goldstein et al. (2022) showed that both human brains and a new type of deep language model (which is designed to generate appropriate linguistic responses for a given context) continuously predict the next word when processing an ongoing narrative. Of direct relevance to the current study, Saalasti et al. (2019) used Word2Vec to calculate the similarity in how participants interpreted a narrative and then showed that this correlated with ISCs in fMRI brain activity.

There are a number of outstanding questions not addressed by previous studies of narrative processing. First, Keidel et al. (2018) manipulated whether their video clips showed a continuation of the same event or two completely different events, so their results may not solely reflect processing of the dialogue. Second, no study has investigated how the semantic content of recalled descriptions of events is influenced by the provision of prior knowledge about the events. This, combined with ISC analyses of fMRI data, will allow us to investigate the proposal that prior knowledge constrains how semantic information is activated and encoded in particular situations. This proposal predicts that when individuals share the same prior knowledge about an event, their neural processing of the event will be more synchronized and the semantic content of their recollections of the event will be more similar.

To address these gaps in the literature, we conducted two separate experiments that used a design similar to that of Keidel et al. (2018). We used scenes from TV sitcoms separated into two parts. The part 1 clips were either presented normally [normal-speech (NS)] or with spectrally rotated speech (SRS) to render the dialogue incomprehensible. The part 2 clips always showed the conclusions of the events and were presented with normal sound. Both conditions clearly depict the same event, and participants can readily perceive the location, the characters present, and their actions and emotions. Thus, the key difference between the part 2 conditions [high-context (HC) vs low-context (LC)] is the knowledge of the specific content of the first half of the conversations.

The first experiment was a large-scale behavioral experiment conducted online, where participants recalled the part 2 video clips shortly after watching them. Key analyses focused on the intersubject similarity of the recalled descriptions when encoded in multidimensional semantic space. The second experiment used fMRI; participants watched all videos in the scanner and then completed a postscan memory test and behavioral ratings. The main analysis of interest was the influence of prior knowledge on ISC of brain activity during the part 2 video clips. In both experiments, we hypothesized that knowledge of the content of the preceding dialogue would lead participants to process and remember the second parts of the conversations in a more coherent and consistent manner. This would increase alignment across participants, not only in their neural processing, but also in the content of their recalled descriptions. We expected prior knowledge to modulate processing most in regions closely associated with semantic processing.

## Materials and Methods

### Participants

Experiment 1 was conducted online and recruited 207 participants (42 males, 165 females) who were undergraduate psychology students fluent in English, but were not necessarily native English speakers. Participants had a mean age of 19.61 years (SD, 2.0). We excluded 38 participants from the study because they failed to recollect >30% of the videos used in the study. Therefore, 168 participants were included in the main analysis. Participants received course credits in exchange for their participation. The experiment was approved by the Research Ethics Committee, and all participants gave informed consent and received course credits for participation.

Experiment 2 involved fMRI and recruited a new cohort of 24 right-handed native English speakers with a mean age of 22.26 years (SD, 3.12; 6 males) and with normal or corrected-to-normal vision. The sample size was based on prior work using ISC and comparing the effects of prior knowledge on the processing of naturalistic stimuli (Lahnakoski et al., 2014; Ames et al., 2015; Saalasti et al., 2019). Four participants were not included in the final analysis because of artifacts in the MRI scans and one further participant did not complete the experiment. One participant had corrupted postscanning behavioral data for the memory questions and was not included in the behavioral analysis. The project was approved by the Research Ethics Committee, and all participants gave informed consent and were paid £20.

### Stimuli

Twenty scenes from different US and UK television shows were used in the experiment. The shows originally aired between 1970 and 2003 and were selected to be unfamiliar to our sample. Each scene was divided into two video clips of approximately equal lengths; part 1 and part 2. The location and characters remained constant across the two parts. For our main experimental manipulation, the speech in 10 of the part 1 video clips was made unintelligible. This was done by spectrally rotating the audio of the videos with a sinusoidal function with maximum frequency of 4 kHz using Praat (version 6.0.15; Boersma, 2001). This keeps the intonation and rhythm of the speech but makes it incomprehensible. The audio for all videos was scaled to have the same mean decibel intensity. The mean duration of all the excerpts was 32.47 ± 3.88 s. The part 1 video clips (30.57 ± 4.38 s) were on average shorter than the part 2 video clips (34.37 ± 2.02 s). Three different videos, all with spectrally rotated audio, were used in the practice task.

### Procedure

The same basic procedure was used for experiment 1, which was run online, and experiment 2, which was run in the MRI scanner. Experiment-specific details are given in the following section. Participants were informed that they would be shown scenes from TV sitcoms each split into two parts. They were told that the speech in half of the part 1 video clips would be unintelligible and were asked to watch the clips as they would watch television at home. Participants were also informed that their memory for the video clips would later be tested. They all then completed a 6 min practice session before starting the experiment, to familiarize themselves with the task and the sound of spectrally rotated speech.

**Figure 1.**
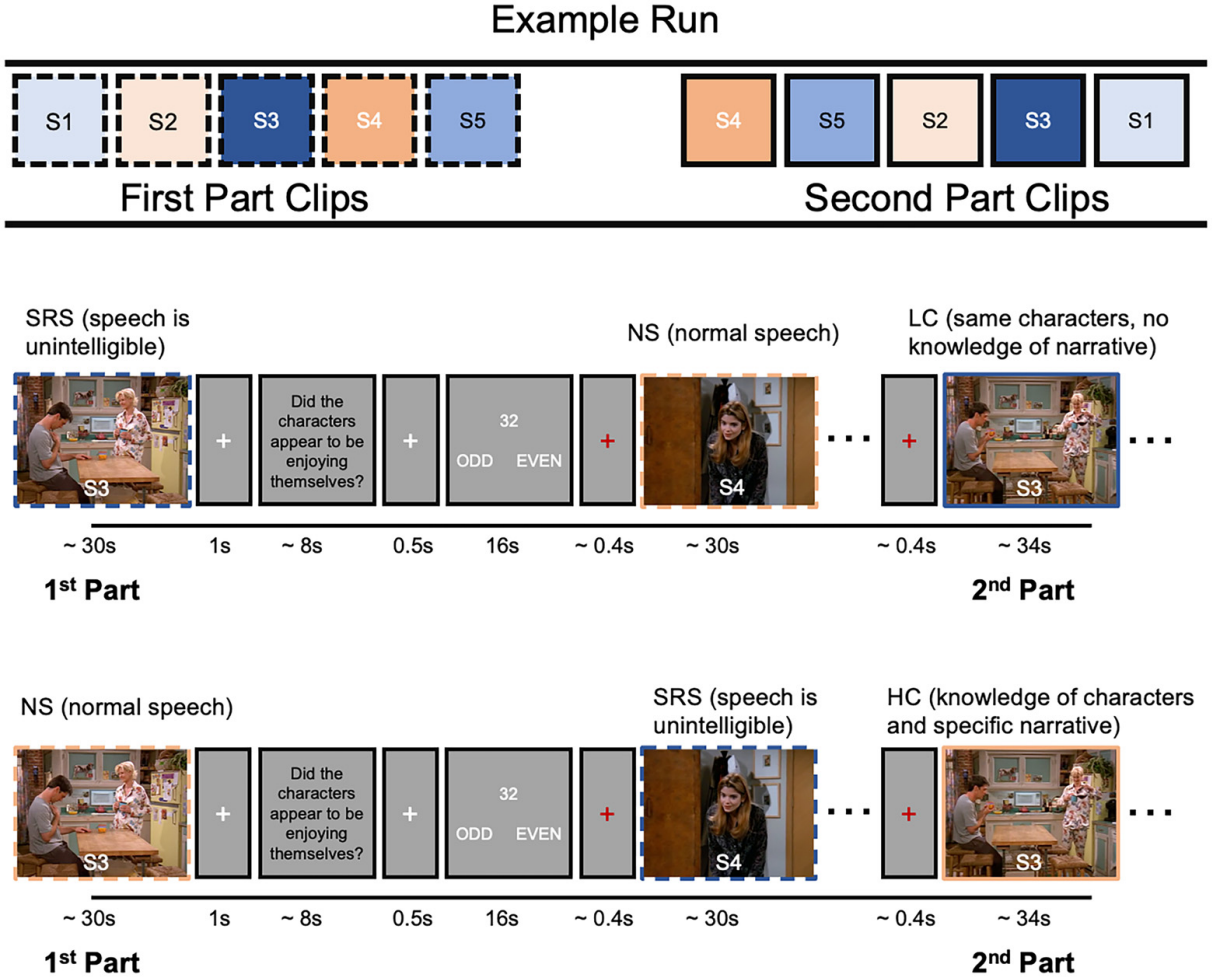
Schematic of study design. Participants viewed clips from unfamiliar TV sitcoms that were divided into two parts. Top row shows the example order of stimuli (e.g., S1, show 1; S2, show 2). Blue designates LC pairs of part 1 and part 2 clips; orange designates pairs of HC clips. Participants viewed a set of 5 part 1 clips followed by a set of 5 corresponding part 2 clips presented in random order. Ten of the part 1 clips had comprehensible speech, whereas the other 10 had unintelligible speech, created by spectrally rotating the audio. Clips were counterbalanced across participants in a within-subjects design (see middle and bottom rows). All main analyses were on the part 2 clips, which were identical across conditions and differed only in whether participants had knowledge of the preceding (part 1) dialogue (HC condition) or did not (LC condition).

The design is shown in Figure 1, and an example of the task is available at https://tinyurl.com/3b42fzzd. Each participant watched 10 part 1 clips with normal speech (NS) and 10 part 1 clips with SRS. Importantly, watching a part 1 clip with SRS still provided a lot of information about the continuation event. Thus, although the dialogue between the actors was incomprehensible, the locations, people present, their actions, and the emotional tone of the event were still obvious (e.g., participants would be able to see that a video depicted two smartly dressed women having a convivial conversation at a table in a café). All 20 part 2 clips were presented with normal speech. The part 2 clips that correspond to the part 1 clips that were shown with normal speech are designated HC, as the participant had full knowledge of the content of the preceding dialogue. The corresponding part 2 clips to those seen in part 1 with spectrally rotated speech are designated as LC. Note that although the speech in the part 2 clips from the LC condition is meaningful and comprehensible, participants do not have any knowledge of the content of the preceding dialogue. The designation of each video to the HC/LC condition was counterbalanced across participants, as was the order of presentation of the videos within each list, resulting in four separate counterbalanced orders of stimuli presentation.

Participants watched part 1 and part 2 video clips in four runs of five videos per run. Each run was ∼10 min long and consisted of five part 1 and five part 2 video clips (Fig. 1). To verify that participants were attending to the clips, each one was followed by a question about the general relationship between the characters in the scene (e.g., “Did the characters appear to be enjoying themselves?”). Behavioral piloting confirmed that participants could answer the questions regardless of whether speech was rotated or not. The questions were presented for 8 s or until participants made a yes/no response. Each question was then followed by a simple numerical task lasting ∼16 s. This ensured that participants were not rehearsing the clips during the intertrial intervals and provided an “active baseline” for the fMRI experiment (Binney et al., 2016). Six randomly chosen numbers, between 1 and 98, were sequentially presented for 2 s, and participants made odd/even number judgements. White fixation crosses were presented after each video clip offset (1 s), after a yes/no response was made to each question (0.5 s) and between each number presentation (0.67 s). A red fixation cross was presented after the numerical task for a minimum of 0.4 s to indicate that the next video clip was about to begin. Within a run, all five part 1 video clips were presented first, followed by the set of corresponding part 2 video clips in random order. This meant that a part 2 clip did not immediately follow its corresponding part 1 clip. In each run, there were either two or three clips from the HC condition.

Memory testing and other subjective ratings were only conducted for the part 2 video clips. For each part 2 clip, the first 4–6 s were presented as a memory cue. Participants were then asked to rate the following: (1) the vividness of their memory of the video clip; (2) how coherent they found the story in the video clip; and (3) how engaging they found the video clip. Participants were also asked one short-answer open-ended question about a detail from the video clip (e.g., “What was the address on his chest written in?”; answer: “lipstick”). Responses were scored as either correct or incorrect.

### Procedural details specific to experiment 1

Experiment 1 was conducted online using jsPsych (https://www.jspsych.org). After the four encoding runs during which the videos were shown, participants were presented with a screen indicating that testing would begin. Memory for the videos was cued and tested in a random order, and the same ratings and questions were used as detailed above. The rating scale ranged from 1 to 100. In addition, after the ratings were completed, participants were asked to type into a free text box what they could remember about the remainder of the video clip.

### Procedural details specific to experiment 2

The video-encoding runs were conducted in the MRI scanner. The scanning session started with a 4 min resting-state scan (data not reported here). Video clip onsets were all time locked to the start of a brain volume acquisition. Memory testing and subjective ratings were conducted immediately after the scanning session in a quiet room using the experimental procedure detailed above. The rating scale ranged from 1 to 10. In addition, participants completed a questionnaire about their familiarity with the TV shows used in the study. Only six participants reported any familiarity with 1–3 of the 20 shows. This represented only 3.9% from the data used in the analysis. None of the participants were familiar with the particular scenes used in the experiment.

### MRI acquisition

Data were acquired on a 1.5 T scanner (Avanto MRI scanner, Siemens). Functional T2*-weighted BOLD-sensitive images were acquired with an EPI sequence with the following parameters: FOV, 192 mm; TR, 2.62 s; TE, 42 ms; 90° flip angle; slice thickness, 3 mm; 35 interleaved ascending slices with 0.6 mm gap; and 3.0 × 3.0 × 3.0 mm voxels. A high-resolution T1-weighted image [MPRAGE (magnetization-prepared rapid acquisition gradient echo)] was acquired with the following parameters: FOV, 256 mm; TR, 2.73 s; TE, 3.57 ms; 1.0 × 1.0 × 1.0 mm voxel size.

### Image preprocessing

All EPI images were preprocessed using SPM 12 (Wellcome Department of Imaging Neuroscience, University College London, London, UK). Field maps were used to correct for image distortions and susceptibility-by-movement effects using the Realign and Unwarp option (Andersson et al., 2001). All EPI images were aligned to the first image of the first session. The anatomic image of each subject was coregistered to their mean realigned EPI images. The anatomic images were then segmented into gray and white matter maps. Anatomical and EPI images were normalized to the MNI space using DARTEL (Ashburner, 2007) and smoothed with an 8 mm FWHM kernel.

### Behavioral data analysis

Behavioral data were analyzed using R, and mixed-effects models were fitted using the lme4 (Bates et al., 2015) and lmerTest packages.

We fitted separate linear mixed-effects models for the subjective memory ratings in experiments 1 and 2. We used condition as a fixed effect and allowed its slope to vary across participants and videos, and we also included a random intercept for subject and video [e.g., Vividness ∼ Condition + (Condition | Subject) + (Condition | Video)]. Note that, because of convergence issues in experiment 2, we dropped the random slope term. We used logistic mixed-effects models for the free-recall trials in experiment 1 and the short memory questions in experiment 2. We used the logit link function and modeled the responses as coming from a binomial distribution. We used condition as the fixed effect with random slopes and intercepts for subject and videos [e.g., Memory_Question ∼ Condition + (Condition | Subject) + (Condition | Video)].

For the free-recall data collected in experiment 1, we scored each trial as either remembered or not. Trials on which participants did not provide any correct details of the video or did not provide information that was not present in the memory cue video were rated as not remembered and scored 0. Trials on which participants correctly remembered the gist of the clip and provided some details about the clip were scored 1 (e.g., “The woman in the clip claims that penguins cannot fly because their wings cannot support their body-mass. The man disagrees and claims that the sky used to be filled with penguins.”). Importantly, the free-recall data allowed us to examine whether participants provided more consistent descriptions for the HC videos when compared with the LC videos. We used the Google Universal Sentence Encoder (USE; Cer et al., 2018) to examine semantic similarity for remembered trials in the HC and LC conditions. First, we selected only remembered trials for the HC and LC conditions. We then converted the memory responses of participants into vectors of 512 numbers using the USE. The USE embeds sentences into a multidimensional semantic space and allows the distances between descriptions to be quantified (Heusser et al., 2021).

After embedding each recollected trial into this semantic space, for each video we extracted the recall responses that were given by participants who saw the video in the HC condition and by the other participants who saw the same video in the LC condition. For example, for video 1, 74 participants correctly remembered it and had seen it as HC video, and another 71 participants correctly remembered it and had seen it as LC video. For each video, we created two average semantic vectors, one for the HC condition and one for the LC condition. We then computed the similarity between each participant’s embedded recall description and the average description of the same video seen in the same condition. We used the Pearson correlation coefficient to compute similarity between the embedded recall descriptions. Note that the average descriptions were always recalculated to not include the description of the current participant (using a “leave-one-subject-out” procedure). For instance, for video 1, we would compute the similarity between the response of participant 1 for that video and the average responses of all other participants who remembered that video in the same condition. We then Fisher transformed and averaged these similarity scores across participants to result in two average consistency scores per video (one for the HC condition and one for the LC condition). We then compared for each video for whether participants on average provided more consistent responses for the HC condition than for the LC condition using a nonparametric permutation test. We flipped the sign of the difference between HC and LC videos 5000 times, which is effectively permuting the conditions the videos are in. This allowed us to construct a null distribution with which to compare the observed HC and LC difference and obtain a *p*-value.

### fMRI data analysis

Data were analyzed with SPM 12, the CoSMoMVPA toolbox (Oosterhof et al., 2016), and custom scripts in MATLAB (version 2016b; MathWorks). Permutations tests for whole-brain analyses were conducted with command-line functions in FSL (Nichols and Holmes, 2002; Winkler et al., 2014). All analyses were conducted on MNI normalized images within a gray matter mask. Segmentation of the high-resolution structural images provided us with gray matter tissue probability map for each subject. These probability maps were normalized to MNI, averaged across participants. The averaged mask was smoothed with an 8 mm FWHM kernel. We selected all voxels within this average probability map higher than a threshold of 0.3 (Nastase et al., 2019). To describe and visualize our data, we used the Bspmview toolbox (www.bobspunt.com/bspmview), which implements the MNI coordinates from the Anatomical Automatic labeling 2 toolbox for SPM 12. Significance was tested with a one-sample random-effects *t* test against zero. Unless otherwise stated, images were whole-brain cluster corrected for FWE at *p* < 0.05 at a voxel height-defining threshold of *p* < 0.001.

#### Univariate general linear model analysis

A single task regressor for each of the four conditions (SRS, NS, LC, and HC; Fig. 1) was included in the first-level models. For all general linear model (GLM) first-level models, the questions after each video were modeled with a single regressor of no interest and the odd/even number judgment task was left unmodeled to represent the implicit baseline. A block design first-level analysis was conducted to replicate previous findings. In this analysis, all video stimuli were modeled with boxcar functions whose durations matched the stimulus duration. The models also included the six motion parameters, a regressor for the mean session effects, and a high-pass filter with a cutoff of 1/128 Hz. Within predefined regions of interest (ROIs), we ran a finite impulse response (FIR) model to examine the condition average activation time course for the HC and LC videos. This enabled us to replicate the analyses of Keidel et al. (2018).

#### Intersubject correlation analyses

We conducted two separate ISC analyses. The first investigated regions in the brain whose fMRI time courses of activity were synchronized while watching the same video clips regardless of whether participants had prior knowledge of the preceding dialogue or not. We refer to this as the “general ISC effect.” Next, we identified those regions where ISCs were higher in the HC condition video clips compared with the LC condition video clips. This second analysis identifies regions that are not only engaged when watching the videos, but also where activity is modulated by the availability of prior knowledge about the conversations that are being watched.

ISCs were computed voxelwise within a whole-brain mask over the part 2 video clips. The raw time course for each video clip and each subject was extracted. These time courses were used to compute the Fisher-transformed correlations across subjects for each video. The first two TRs (5.24 s) of each video were removed to remove transient onset effects that can lead to artificially high ISCs (Ames et al., 2015).

To examine the general ISC effect, for each participant and each video we computed the following: (1) the correlation between their own video clip-specific time course and the average time course for the rest of the participants watching the same video clip; and (2) the correlations between their own video clip-specific time course and the average time course for the rest of the participants watching the other video clips shown within the same run (e.g., correlating the time course of a subject watching “Dharma and Greg” with the average time course of other subjects watching “Just Shoot Me”). This resulted in a total of 100 correlations of interest per participant: 20 ISCs while watching the same video clip, and 80 correlations while watching different video clips. The key contrast is the difference in ISCs when watching the same video clip versus the ISCs when watching different video clips (see Fig. 5, contrast matrix used for this analysis). This general ISC analysis replicates previous work that has examined synchronization across participants watching the same movie or video clips.

To investigate the influence of prior knowledge on ISCs while watching the video clips, it was necessary to construct two different condition lists (since the HC and LC condition video clips were counterbalanced across participants). There were 9 and 10 people in the two condition lists. For each participant, we computed the correlation between their own video clip-specific time course and the average time course for the rest of the participants in the same condition list watching the same video clip. This resulted in 20 time course correlations for each participant: 10 from the HC video clips and 10 from the LC video clips. For this analysis, the key contrast was for the correlations from the HC video clips versus the LC video clips (see Fig. 5 for the contrast matrix used for this analysis).

For both analyses (general ISC and prior knowledge ISC), the raw correlations were Fisher transformed before the contrasts of interest were computed. The contrasts were conducted for each participant, resulting in 19 brain maps of the difference in correlations that were used in the group analyses. Since these brain maps are not necessarily independent (Aly et al., 2018), we used nonparametric permutation tests to compute the significance at the group level. The difference in Fisher-transformed ISCs between conditions was computed for the general and prior knowledge ISC analyses separately. To perform the permutations, the sign of the resulting difference was flipped for a random subset of subjects before computing the group mean. This effectively is the same as shuffling the conditions for different subjects. The 5000 permutations were run (per analysis) to obtain the null distribution with which to compare our observed data and obtain *p*-values. Cluster-corrected images at an FWE of *p* < 0.05 at a voxel height-defining threshold of *p* < 0.001 are presented in Figure 5.

### Supplementary analyses

We additionally conducted ISC analyses of the data from the part 1 video clips to identify brain regions that are more synchronized across participants watching videos with intelligible speech, compared with unintelligible speech. These analyses do not address our experimental hypotheses but are presented in supplementary materials for completeness (see https://osf.io/f9jd4/).

We also conducted exploratory intersubject analyses using the spatial patterns of brain activity [intersubject pattern similarity (ISPS)] averaged over the whole of the video clips, rather than the temporal ISC. These were conducted for the same contrasts as the ISC analyses described above. Full details of the methods and results of these analyses are described in the supplementary materials (see https://osf.io/f9jd4/).

Finally, based on recent studies examining reinstatement effects in hippocampus when participants were listening to stories referring to previously heard information (Chang et al., 2021; Cohn-Sheehy et al., 2021), we examined whether we would observe higher ISC or ISPS effects in hippocampal ROIs for the HC video clips when compared with LC video clips (see https://osf.io/f9jd4/).

## Results

### Behavioral results

**Figure 2.**
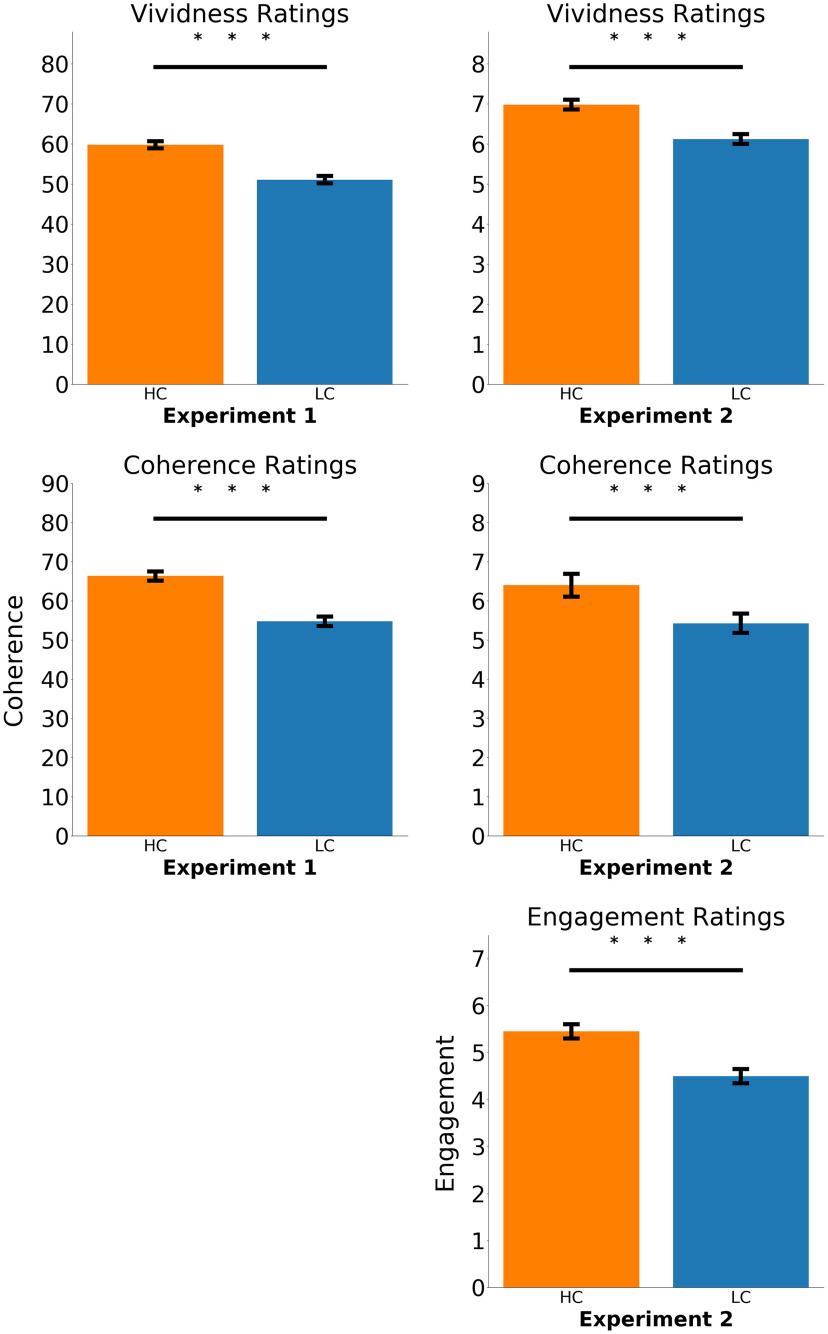
Subjective memory ratings were better for the HC videos. Left column shows results from subjective measures in experiment 1, and right column shows subjective measures in experiment 2. Error bars represent standard error of the mean. **p < 0.01, ***p < 0.001.

Participants in experiment 1 rated their memory for the HC videos as more vivid compared with the LC clips (59.75 vs 51.04; *t*_(24.02)_ = 6.62, *p* < 0.001). Participants also found the HC videos to be more coherent (66.33 vs 54.78; *t*_(65.61)_ = 8.79, *p* < 0.001). Similarly in experiment 2, participants rated their memory for the HC compared with the LC videos to be more vivid (*t*_(323.04)_ = 4.42, *p* < 0.001). They also found the HC versus the LC videos to be more coherent (*t*_(324.86)_ = 4.83, *p* < 0.001) and more engaging (*t*_(323.86)_ = 4.62, *p* < 0.001; Fig. 2).

**Figure 3.**
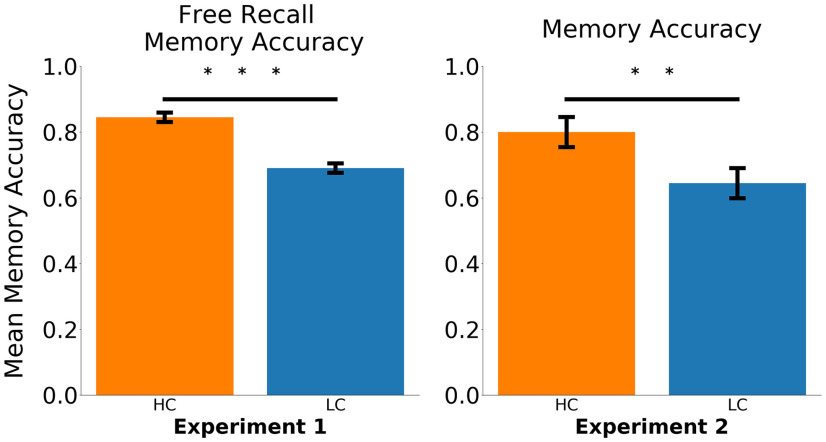
Participants remembered the HC videos better. We show that free-recall accuracy was higher for the HC videos compared with the LC videos. Participants in experiment 2 showed better memory, tested with open-ended memory questions, for the HC videos compared with the LC videos. Error bars represent standard error of the mean. **p < 0.01; ***p < 0.001.

**Figure 4.**
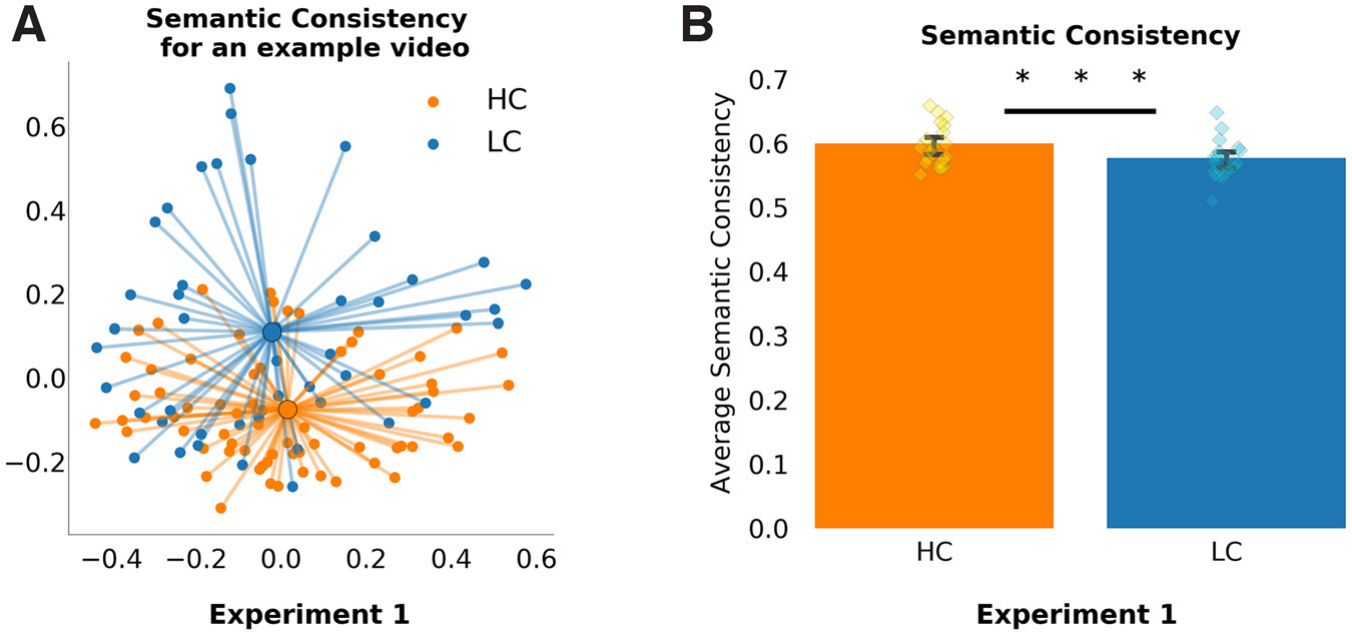
Semantic consistency is higher among recall of HC videos. ***A***, A PCA projection in 2 dimensions of the semantic vectors that represent all participants’ correct free-recall responses for a single video. Semantic consistency for each video was calculated within each condition. The larger center dots are the condition average semantic patterns for the video based on the free-recall responses. Lines toward the center are for illustrative purposes. ***B***, We calculated the average spread of scores for each video under each condition. One data point in the bar graph represented the average spread of responses across participants watching a video in one condition (e.g., average spread in orange points in ***A***). Semantic consistency was on average higher for the HC videos. Error bars represent 95% confidence intervals. ***p < 0.001.

Participants freely recalled on average 76.8% of the video clips and performed better on the HC videos when compared with the LC videos (84.35% vs 69.4%; *Z* = 6.76, *p* < 0.001). In experiment 2, participants were able to answer 72.2% of the memory questions, which is a high level of performance, given the fact that the questions were open ended (Fig. 3). Participants responded more accurately to the same questions when in the HC condition than in the LC condition (80% vs 64.4%; *Z* = 2.02, *p* = 0.04). The main purpose of the online study was to examine the semantic consistency across participants of their recalled descriptions of second-half clips. We also used the Google USE to quantify the semantic similarity of free-recall responses for the videos in HC and LC conditions. Specifically, we examined how similar individual participants’ free-recall responses were to the group average free-recall responses, and, critically, whether the similarity was higher when the group had watched the video in the HC condition compared with the LC condition. Consistent with the view that the provision of prior knowledge guides and constrains how the content of the concluding part of the dialogue is interpreted and represented, we found that second-half descriptions were more similar across participants when the videos were seen in the HC condition when compared with the LC condition (*t*_(19)_ = 3.71, *p* = 0.001; see Fig. 4).

### fMRI results

#### GLM

The contrast of watching videos versus the active baseline task revealed higher activation in visual, auditory, medial, and anterior temporal cortices (see https://osf.io/f9jd4/, Supplementary Fig. 1). This is consistent with previous studies using videos (Bartels and Zeki, 2004). Contrasting first-half clips with NS versus videos with SRS, identified regions commonly associated with language and semantic tasks (see https://osf.io/f9jd4/, Supplementary Fig. 2. The results of the FIR time course analysis were also consistent with this previous study in showing higher activation in the MTG, supramarginal gyrus, and AG for videos depicting a continuation of a previous narrative (HC clips). For further discussion, see https://osf.io/f9jd4/ (Supplementary Fig. 3 and Materials).

#### Intersubject correlation

**Figure 5.**
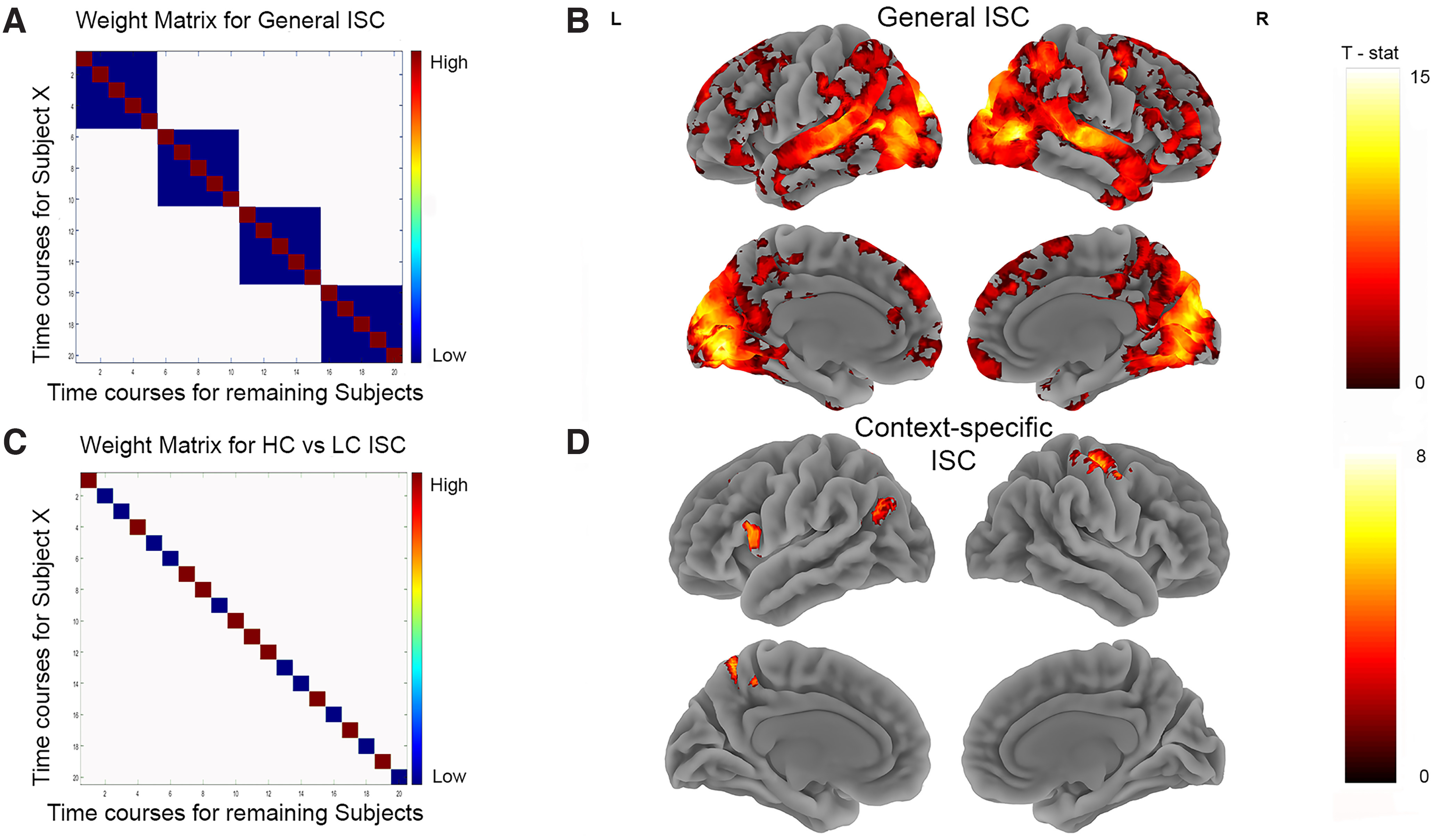
Intersubject correlations. ***A***, The weight matrix (General ISC) tests for video-specific time course similarity across participants. Each cell represents the correlation between subjects’ time course for a particular video with the average time course of all remaining participants for a particular video. The diagonal represents correlations between time courses for the same videos. The off-diagonal represents temporal correlations between mismatching videos within the same run. ***B***, Brain map from video-specific analysis, which shows extended synchronization across the brain for people watching the same videos. ***C***, Weight matrix that tests for the time course similarity across the same videos, depending on their prior knowledge. ***D***, Brain map showing how time course synchronicity was modulated by prior knowledge. Both brain maps show clusters significant at FWE *p* < 0.05 after permutation testing.

The general ISC analysis allowed us to examine synchronization of the BOLD response across participants. ISCs for watching videos regardless of the context manipulation were found in extensive regions of the occipital and superior temporal lobes, encompassing visual and auditory processing regions. Higher synchronization was also observed in bilateral IFG, medial prefrontal cortex, AG, and precuneus (Fig. 5).

The key ISC comparison between HC and LC videos showed stronger coupling in the left AG, IFG, superior parietal lobule, superior frontal gyrus, and right precentral gyrus (Fig. 5). In these regions, the synchrony across individuals is significantly greater when those individuals share knowledge of the preceding narrative.

## Discussion

This study examined the cognitive and neural effects of prior knowledge of a narrative on the processing of conversations between two people. In one behavioral experiment and one fMRI experiment, participants viewed videos of dialogues for which they either were, or were not, provided with knowledge of the first part of the conversation (HC and LC conditions, respectively). Key behavioral results replicated closely across the two studies: prior knowledge resulted in better memory for the dialogues as well as increased subjective ratings of vividness and coherence. In the online behavioral experiment, using automated text-based measures, we showed that across participants, the recalled descriptions of the HC clips were semantically more similar to each other compared with the LC clips. In the fMRI experiment, we identified a number of brain regions that showed higher ISCs in the HC condition, particularly those associated with the semantic network of the brain (including the left superior and inferior frontal gyrus, and left AG).

Our results extend and clarify the findings of Keidel et al. (2018), whose results may have reflected the role of the IFG and AG in supporting neural representations of broader “situation” or “event” models (Ranganath and Ritchey, 2012; Chen et al., 2017; Bird, 2020). We also show that when people recall an event, the semantic content of their descriptions is more aligned across participants who share the same prior knowledge about that event. This finding dovetails nicely with our fMRI results showing higher ISCs when people watch conversations for which they share knowledge about the preceding dialogue. Together, our results support the proposal that listening to a conversation establishes a set of linked semantic concepts, which are reactivated when we pick up the conversation after a break. As a result, our comprehension and memory for the conversation is both boosted and constrained by our knowledge of the preceding dialogue. The IFG and AG appear to play a key role in processing the semantic concepts necessary to understand, comprehend, and remember a conversation. We discuss these conclusions in more detail below.

In both our online behavioral experiments and fMRI experiments, prior knowledge improved cued-recall memory accuracy for details from the part 2 clips. It also increased subjective ratings of how coherent and engaging the part 2 clips were as well as how vividly they could be remembered. These effects are consistent with previous studies (Dooling and Lachman, 1971; Bransford et al., 1972; Johnson et al., 1974; St. George et al., 1994; van Kesteren et al., 2014; Ames et al., 2015; Brod et al., 2016; Liu et al., 2017; Raykov et al., 2020, 2021). Nevertheless, the differences between the HC and LC conditions were not particularly large for any of these measures; in general, all video clips were given quite high ratings for coherence and engagement, and the overall performance on memory questions was good. We conclude from this that prior knowledge likely had a beneficial effect on the comprehension and memory for the clips in the HC condition, rather than the LC clips being particularly confusing or difficult to understand.

Understanding something as complex as a scene from a television sitcom requires the activation and integration of numerous different semantic concepts. Providing that the first part of the conversations provides a framework that can help interpret what is said during the second part. To give an example, in one of the part 2 videos, a character claims that penguins once “filled the skies” but are now unable to fly because they lack the confidence to do so. Participants viewing this clip in the HC condition can relate this discussion back to the first part in which the characters discuss how low self-esteem had a negative effect on a colleague’s work. However, participants in the LC condition do not have this background context to draw on and tend to form more idiosyncratic interpretations of the scene.

Our first experiment used a universal sentence encoder to investigate whether knowing the content of the dialogue from the first half of the video resulted in greater similarity between people’s descriptions of the second half of the videos. This was found to be the case, suggesting that prior knowledge constrained the elements of the events that were recalled to be more similar across participants. Since these effects were seen when recalling the clips after several minutes, we cannot be sure whether the greater alignment across participants happened during recall, or when initially watching the second half clips. However, given that we also observed greater neural ISCs when participants watched the second-half clips in the HC condition, it is likely that prior knowledge constrained how participants interpreted the dialogues and encoded them to memory.

In an earlier study with a similar design, we found that HC clips were associated with increased activity in the AG, IFG, and MTG (Keidel et al., 2018). Our present study replicated these findings for the AG and the MTG (but not the IFG), despite using a more tightly controlled design where the HC and LC first-half videos were identical except for the manipulation of the sound (see https://osf.io/f9jd4/, Supplementary Materials) We additionally found higher ISCs in four brain regions while participants viewed videos in the HC condition, including two regions identified in the earlier study—the left AG and IFG. The fact that activity is more synchronous in these regions across participants who share the same prior knowledge suggests that processing in these regions is modulated by this knowledge. Consistent with the conclusion of Keidel et al. (2018), we suggest that knowing the content of the dialogue from the first half of the conversation constrains the activation of sematic concepts that are most relevant for understanding and interpreting the second-half videos. This results in not only perceiving the clips to be more coherent, but also boosts the alignment of activity in brain regions that process the semantic concepts.

The brain regions where ISCs were modulated by prior knowledge have been particularly associated with “semantic control”—the selection and retrieval of appropriate semantic information in the face of competing but irrelevant information (Binder et al., 2009; Ralph et al., 2017; Gao et al., 2021). A number of recent studies using spoken short stories have also implicated the IFG, AG, and lateral temporal regions in the integration of prior knowledge with incoming information to understand the unfolding narrative (Yeshurun et al., 2017; Chang et al., 2021). More generally, when people interpret a narrative in a similar way, brain activity is more synchronized within these regions (Nguyen et al., 2019; Saalasti et al., 2019). Causal evidence for a role of the AG in the integration of prior information to understand a narrative comes from a recent study by Branzi et al. (2021), who used TMS on the left AG, which resulted in disrupted context-dependent comprehension of prose passages. Together, these studies suggest that the MTG, IFG, and AG support the selection, integration, and representation of conceptual information required to understand dialogues as they unfold over time.

These empirical findings mesh well with recent theoretical accounts of processing with DMN structures. The inferior lateral parietal cortex has been viewed as supporting a “buffer” for episodic information (Baddeley, 2000; Vilberg and Rugg, 2008) or for linking together the features of an event into a cortical representation (but see Shimamura, 2011; Ramanan et al., 2018; Bromis et al., 2021). Recently, Humphreys et al. (2021) argued that the angular gyrus supports “consciously accessible representations that integrate features of events that unfold over time” (but see Hasson et al., 2008, 2015; Lerner et al., 2011). Others have argued that its role is in supporting abstract conceptual representations of events (e.g., a “birthday party”; Binder and Desai, 2011). Whether or not different brain regions play a greater role in the selection of relevant semantic information, versus the maintenance and integration of this information over time, remains a question for future research.

It is possible that our fMRI ISC results are caused by alignment of other cognitive processes beyond semantic processing. Sharing knowledge of the preceding dialogue might lead people to allocate attention to the second-half video in a more consistent manner or engage more similar emotional processes. However, our design aimed to minimize the likelihood of this. The part 1 clips in both HC and LC conditions were visually identical and therefore people can readily see the characters in the videos, what they were doing, their emotional states and where the videos were set. The key information that participants lacked in the LC condition was the content of the part 1 conversations. It remains possible that the contents of the dialogues themselves elicit emotional or empathic responses that differ in the HC and LC conditions. Further research may be able to tease apart the processing of the semantic concepts that comprise a narrative and the emotional responses to that narrative.

How is the information from the part 1 clips stored before it is used to process the continuation of the conversations in the second-half clips? Since we used filled intervals between the first and second halves of the clips, it is unlikely that the information is actively held in working memory, either in the classic sense of a short-term buffer (Baddeley, 1992) or held online in brain regions that integrate information over timescales of up to a few minutes (Hasson et al., 2015). It is possible that the information is held in a “silent” or “latent” working memory state (Stokes, 2015; Sprague et al., 2016; Wolff et al., 2017), where it can be stored for durations of several minutes and then rapidly activated when needed. Alternatively, it could be stored in long-term memory, either explicitly as an episodic memory for the content of the first half, or more implicitly in the form of primed associations between the semantic concepts that are central to understanding the dialogue (Beukers et al., 2021). Unfortunately, it is impossible to tease apart these possibilities using fMRI combined with ISC measures. This technique can only shed light on what processes are currently active, not the source of the information that is being processed. Indeed, fMRI effects that were previously considered to index episodic recollection are now thought to largely reflect semantic processing of the retrieved information (Renoult et al., 2019).

Many studies have highlighted a particular role for the hippocampus in the integration of information held in memory (Shohamy and Wagner, 2008; Zeithamova et al., 2012a, b; Schlichting and Preston, 2015; Backus et al., 2016; Clewett et al., 2019; Griffiths and Fuentemilla, 2020; Zeithamova and Bowman, 2020; Cohn-Sheehy et al., 2021). We therefore carried two exploratory analyses to examine whether ISCs of BOLD activity and intersubject spatial patterns of BOLD activity were more similar within the hippocampus for the HC videos. There was no effect of condition on ISCs, but we did observe higher pattern similarity for the HC videos in the right hippocampus at a marginally significant level (see supplementary analyses: https://osf.io/f9jd4/). Two previous studies have also implicated the right, but not the left, hippocampus, in the integration and differentiation of narrative storylines (Milivojevic et al., 2016; Cohn-Sheehy et al., 2021). Therefore, there is some evidence that the hippocampus plays a role in activating and integrating information from memory to process current experiences.

In summary, knowledge about a preceding dialogue enabled participants to better comprehend and remember the conclusions of these dialogues. The availability of narrative information led to increases in intersubject measures of the similarity of recall of the events and the synchronization of fMRI activity, predominately in regions associated with semantic processing. We argue that prior knowledge constrains the activation of those semantic concepts that are relevant to understanding the unfolding dialogue. This results in tighter coupling across participants of (1) ongoing semantic processing of events as they unfold and (2) the content of what can be later recalled about these events. Our findings highlight a role for the angular gyrus and inferior frontal gyrus in the processing of concepts necessary to understand a complex dialogue. These results provide new insights into how the brain represents and updates narrative information.

## Synthesis

Reviewing Editor: Anne Keitel, University of Dundee Decisions are customarily a result of the Reviewing Editor and the peer reviewers coming together and discussing their recommendations until a consensus is reached. When revisions are invited, a fact-based synthesis statement explaining their decision and outlining what is needed to prepare a revision will be listed below. The following reviewer(s) agreed to reveal their identity: MASAYA MISAKI, Laura Verga.

**Synthesis**

The reviewers agreed that this is an interesting study but had a few major comments and suggestions to improve the clarity of the manuscript.

The main points were that the novelty/advances should be fleshed out more in comparison to the Keidel et al. 2018 study; the interpretation of the ISC results needs to be more careful; and more detail on methods and procedures are necessary.

Please also add a significance statement (120 words maximum). The Significance Statement should provide a clear explanation of the importance and relevance of the research in a manner accessible to researchers without specialist knowledge in the field and informed lay readers. The Significance Statement will appear within the paper below the abstract.

Please see below for detailed comments made by the reviewers.

Advances in the field:

A similar experimental setup was used in a previous study (Keidel et al., 2018), and the present result was also similar to that study, while a different analysis approach (ISC) was used. The interpretation of the ISC result needs to be more cautious. ISC indicates the commonality of the response time-course across participants, not an activation amplitude. The present manuscript needs more consideration about the implication of the ISC result. Semantic coherence analysis, which was novel in this study, was performed for independent data from the fMRI samples, so it did not help interpret the fMRI result very much. Thus, what was the improvement of this study compared to Keidel et al. (2018) and how this study advanced our understanding of the role of semantic memory were not clear.

More than no advance, I would rather say “limited advance”. This is due to the fact that the authors heavily rely on the study by Keidel and colleagues. While they mention that a number of outstanding questions were left open, very often they refer back to that study (or others) to highlight similarities, rather than what their study adds in terms of novelty. I would recommend the authors to rework the relevant parts in introduction and discussion to focus more on the strengths and innovative aspects of their study.

Statistics comments:

I would appreciate to have more details on the procedure and analyses in order to better judge their quality and correctness. The statistics are appropriate, while a bit more detail is required in some parts. For example, in p. 8, ‘non-parametric permutation test’; What was permuted, the number of permutations, etc., should be explained.

Summary comments:

The manuscript entitled “The importance of semantic network brain regions in integrating prior knowledge with an ongoing dialogue” used video clips in two parts, to investigate whether information provided in the video clip would enhance the understanding and memory of the second video. Overall, I find this manuscript interesting, well-conceived, and well written. Yet, I find sometimes difficult to exactly understand the procedure or rationale for certain choices. As such, my suggestions are mostly intended to improve clarity and add details that could facilitate the replication of the results in future studies.

The study setup was interesting to tackle the difficult problem, the role of semantic memory in comprehending a dialogue with natural stimuli. Using ISC to evaluate the effect of context knowledge is also a smart approach to studying the response to a natural stimulus. However, the main issues of this study are that the implication of the result is vague or speculative, and the significance of the study’s findings is not clear for improving our understanding of the role of semantic memory in dialogue comprehension. A similar experimental setup was used in a previous study (Keidel et al., 2018), and the present result was also similar to that study, while a different analysis approach (ISC) was used. However, the interpretation of the ISC result needs to be more cautious. It indicates the commonality of the response time-course across participants, not an activation amplitude. Semantic coherence analysis, which was novel in this study, was performed for independent data from the fMRI samples, so it did not help interpret the fMRI result very much. In sum, what was the improvement of this study compared to Keidel et al. (2018) and how this study advanced our understanding of the role of semantic memory were not clear.

Also, the abstract can be improved. What measurement and concrete results were obtained in this study were not clear in the abstract.

Detailed major comments:

1. Abstract

The abstract describes the task procedure and the overview of the results, but what was specifically examined was not presented. For example, the study was composed of two independent experiments, and the inter-subject semantic similarity was evaluated for the first data with a similarity analysis of the Universal Sentence Encoder. The fMRI result was obtained from another data (N=19) with the inter-subject correlation analysis. I understand the word limit of the abstract is tight (<250), but there is still room to make it more informative (the present word count is 189).

2. Significant statement that would be required for eNeuro submission is not provided.

https://www.eneuro.org/content/preparing-manuscript#organization

3. p. 3, ‘There are a number of outstanding questions ...’

This part claims the improvement of the present study compared to previous studies, including Keidel et al. (2018). However, how the first issue was resolved in the present study is not clear. Indeed, in the Discussion (p. 17, ‘How is the information ...’), the authors seemed to admit that they could not address the issue either with a sequential or blocked presentation of two clips.

Regarding the second issue, although adding the text-based measure is a merit of this study, they were examined on independent data set, so its results are difficult to be interpreted in conjunction with the fMRI data (e.g., a correlation between the semantic similarity and ISC).

4. p.3, ‘In an online ...’, p. 4, ‘Experiment 1’.

It should be clarified that there were two independent experiments with different participants in the last paragraph of the Introduction. Otherwise, it is not clear at the beginning of the ‘Participants’ section what Experiment 1 and Experiment 2 were.

5. It is unclear to me when exactly the memory task was conducted. At some point is it said it was immediately after watching the clips, yet in the discussion it is mentioned that it was one week after (at least for the behavioural study). Please clarify.

6. Participants:

(A) were they native speakers?

(B) The sentence “the overall findings were consistent whether the 38 participants were included” is highly confusing. If the participants did not recollect more than 30% of the videos, the fact that including their results did not change the results at all may invite the question whether the results truly reflect what the authors want to investigate. I would either remove this sentence entirely, or qualify it with additional information (e.g., which analysis are referred to?)

(C) For the fmri study, it is said that the sample size was based on previous work. I assume this refers to the 24 original participants. How is this affected by the removal of 6 participants (if I understand this part correctly)? Ie., would the authors say that 18 participants still represent an optimal sample size based on previous work?

7. p. 6, ‘Only 6 participants reported ...’

With this small sample size study (N=19), even a few outlier values could significantly affect the results. Excluding the samples of the clips of these participants might be better to avoid such adverse effects. Or the authors should check the effect of these participants’ responses on the results to confirm their no harmful effects.

8. Stimuli: It is said that the clips were selected to be unfamiliar to participants. Yet:

(A) how did the authors decide beforehand which clips may be unfamiliar?

(B) this is contradicted later, where it is said that 6 participants were familiar with the clips. Did the author check whether these participants’ scores differed from the rest of the group?

9. Behavioral analyses: I am not sure I understand whether the authors used linear or generalized linear mixed effect models; if the latter, they should rather refer to glmer, but most importantly they should then specify which distribution family they used and why; this is rather important as some of their dependent variable were dichotomous if I am not mistaken (correct / incorrect?)

10. Results: I am confused about the difference in scale from exp 1 to exp 2: were the ratings not on a scale from 1 to 10?

11. p. 15, ‘our results highlight a role of the semantic network ....’

The present study cannot delineate the functional implication of the ISC result. ISC is a measure of commonality of signal time-series between participants. Its increase only suggests that the participants responded similarly to the stimuli in regions such as IFG and AG. For example, it could be the case that these regions were more active in variable timings for the LC stimuli to guess their context, while they were less active and stable for HC stimuli. If the high variable activation was the cause of decreased ISC for the LC, we could not argue that the identified regions were associated with semantic memory usage. The phenomenological link between semantic coherence in Exp 1 and ISC does not automatically implicate semantic memory recall in the HC condition; variability in LC clip comprehension is natural because of the lack of contextual information, and such variability may reduce ISC. Thus, differences in ISC may be due to LC activation rather than HC.

12. p. 16, ‘Taken together, these studies suggest that the MTG, IFG and AG support ....’

These processes should be included in both conditions, so this argument could be consistent also with the idea that LC stimuli require more cognitive resources to understand the dialogue. Then, a response to LC stimuli may have increased at different timings for different participants, possibly decreasing ISC.

I understand this is just speculation similar to the authors’ argument, but we should be aware that the ISC cannot provide any functional implication in the current result. Thus, the Discussion needs to be more cautious, avoiding conclusive functional claims.

13. While the outstanding questions were raised in the Introduction (refer to my comment 3), how they were resolved in the present study is not clear. They might be addressed implicitly by the results, but I would like the authors to explicitly indicates the questions and the importance of the answers addressed in this study? That could help clarify the significance of this study over the previous research.

14. p. 16, ‘However, by using identical video clips ...’

The authors argued against the interpretation that attention differences between LC and HC could affect the result. However, their claim is about the first clip, and how their argument is applied to the second clip is not clear. If the participants could extract the context in both LC and HC conditions, that would also negate the interpretation of the semantic memory difference between the conditions.

15. Discussion: I would consider moving the results concerning the hippocampus in the main manuscript instead of in the supplementary materials; at any rate, I would add a figure for this result at least in the supplementary material

Detailed minor comments:

- p2: “It is assumed ... in similar ways” - This sentence is quite vague and redundant in its content (for example, similar is repeated multiple times). Please rephrase.

- p. 3, ‘Experiment 2 ...’

The sex composition of the participants should be provided like in Experiment 1.

- Figure 1., p. 6, ‘Within a block, all five ...’

The figure looks like the corresponding clips of the first and second half were presented in a sequence, and a single context (SRS or NS) was used in one scan. However, in the present experiment, multiple first clips, including both SRS and NS, were presented in random order, and then multiple second clips were presented. The figure could be improved to communicate the task procedure more accurately.

- p. 5, ‘Participants ... in four blocks... Each block was approximately 10 minutes ...’

The term ‘block’ was used for indicating a one scan run. This could be confusing for many readers as the ‘block’ is usually used for a task design in a run (e.g., block-design) in neuroimaging. Using ‘run’ or ’scan‘ may be better in this context.

- Procedure: for the interference task, why were specifically 6 numbers used?

- Behavioral analyses: Lmer package: lme4?

- Fmri analyses:

(A) The main subtitle (fMRI data analysis) is virtually the same as the one following (functional MRI data analyses). Would it be possible to provide a better differentiation?

(B) Analysis with gamma function instead of duration: please explain this was necessary

- p. 6, ‘A high resolution T1-weighted image was acquired ...’

Please provide the scan sequence for the T1w image (MPRAGE?).

- p. 7, ‘We used generalised mixed effect model ...’

Please provide what distribution (or link function) was used in the model.

- p. 8, ‘We than compared ..’

Typo. It should be ‘We then ..’

- p. 8, ‘non-parametric permutation test’

Would you provide a bit more detail about the test (what was permuted, the number of permutations, etc.)?

- p. 8, ‘Univariate General Linear Model (GLM) analysis’

The rating event was not modeled in the analysis. This means that brain response at rating was included in the baseline, which could bias the estimate of the task response. Anyhow, this would not be a significant issue of the present study as this could affect only the response estimates for the first clips, which was not the main result of this study.

- p. 8, ‘Next, we identified those regions were ISCs were ...’

Typo. It should be ‘regions where ISCs were ...’

- Fig. 4,

Is this the result of Experiment 1? The number of points shown in Fig 4B looks too few (N = 207 in Exp1?).

- p. 13, ‘The key ISC ...’

Please indicate the coordinates of these regions.

- Supplementary Materials: I don’t understand Figure 4. Why do the authors deem necessary to report results from Keidel et al in their supplementary material?
